# The Antioxidant Therapy: New Insights in the Treatment of Hypertension

**DOI:** 10.3389/fphys.2018.00258

**Published:** 2018-03-21

**Authors:** Daniela Sorriento, Nicola De Luca, Bruno Trimarco, Guido Iaccarino

**Affiliations:** ^1^Dipartimento di Scienze Biomediche Avanzate, Università Federico II, Napoli, Italy; ^2^Dipartimento di Medicina, Chirurgia e Odontoiatria, Università degli Studi di Salerno, Baronissi, Italy

**Keywords:** hypertension, oxidative stress, nitrosative stress, antioxidants, ROS

## Abstract

Reactive oxygen species (ROS) and reactive nitrogen species (RNS) play a key role in the regulation of the physiological and pathological signaling within the vasculature. In physiological conditions, a delicate balance between oxidants and antioxidants protects cells from the detrimental effects of ROS/RNS. Indeed, the imbalance between ROS/RNS production and antioxidant defense mechanisms leads to oxidative and nitrosative stress within the cell. These processes promote the vascular damage observed in chronic conditions, such as hypertension. The strong implication of ROS/RNS in the etiology of hypertension suggest that antioxidants could be effective in the treatment of this pathology. Indeed, in animal models of hypertension, the overexpression of antioxidants and the genetic modulation of oxidant systems have provided an encouraging proof of concept. Nevertheless, the translation of these strategies to human disease did not reach the expected success. This could be due to the complexity of this condition, whose etiology depends on multiple factors (smoking, diet, life styles, genetics, family history, comorbidities). Indeed, 95% of reported high blood pressure cases are deemed “essential hypertension,” and at the molecular level, oxidative stress seems to be a common feature of hypertensive states. In this scenario, new therapies are emerging that could be useful to reduce oxidative stress in hypertension. It is now ascertained the role of Vitamin D deficiency in the development of essential hypertension and it has been shown that an appropriate high dose of Vitamin D significantly reduces blood pressure in hypertensive cohorts with vitamin D deficiency. Moreover, new drugs are emerging which have both antihypertensive action and antioxidant properties, such as celiprolol, carvedilol, nebivolol. Indeed, besides adrenergic desensitization, these kind of drugs are able to interfere with ROS/RNS generation and/or signaling, and are therefore considered promising therapeutics in the management of hypertension. In the present review we have dealt with the effectiveness of the antioxidant therapy in the management of hypertension. In particular, we discuss about Vitamin D and anti-hypertensive drugs with antioxidant properties.

## Oxidative and nitrosative stress: physio-pathological implications

Reactive oxygen species (ROS) are produced in several cellular systems within the cell: plasma membrane, cytosol, peroxisomes, mitochondria, lysosomes and endoplasmic reticulum (Di Meo et al., 2016). The enzymes involved in ROS generation are: nitric oxide synthase, peroxidases, NADPH oxidase, NADPH oxidase isoforms (NOX), xanthine oxidase (XO), lipoxygenases (LOXs), glucose oxidase, cyclooxygenases (COXs), and myeloperoxidase (MPO) (Bhattacharyya et al., 2014). Moreover, exogenous sources of ROS also exists that include air pollution, smoking, ionizing radiations, foods and drugs, chemical agents, heavy metals, organic solvents, pesticides (Bhattacharyya et al., 2014). ROS derive from oxygen reduction which produces, through several steps, important intermediate products: superoxide anion, hydrogen peroxide, and hydroxyl radical. Superoxide anion (${\text{O}}_{2}^{•-}$) is the most common ROS, which is generated in mitochondria by the electron transport chain (ETC) through the partial reduction of oxygen (Bolisetty and Jaimes, 2013). Superoxide dismutase (SOD) is responsible of H_2_O_2_ production from superoxide anion by means of amino acid and xanthine oxidase or a dismutation reaction. In the presence of metal ions and superoxide anion, H_2_O_2_ can produce the hydroxyl radical (·OH), that is the most reactive and dangerous one (Quinlan et al., 2013; Ogun, 2015).

RNS derives from nitric oxide (NO) that is generated during the breakdown of arginine to citrulline by the NADPH-dependent enzyme nitric oxide synthase (Drew and Leeuwenburgh, 2002). NO is a neurotransmitter and a blood pressure regulator; it is a free radical but is not a very reactive one. NO is able to form other nitrogen reactive intermediates (nitrate, peroxynitrite, and 3-nitrotyrosine), which affect cell function (Ramchandra et al., 2005; Ogun, 2015). NO competes with O2 for the binding at the binuclear center of cytochrome *c* oxidoreductase, leading to the inhibition of cytochrome c oxidase activity (Cleeter et al., 1994). In mitochondria it increases the production of ROS and RNS which affect several processes such as mitochondrial biogenesis, respiration, and oxidative stress (Bolisetty and Jaimes, 2013; Ogun, 2015). NO reacts with ${\text{O}}_{2}^{-}$, which derives from mitochondrial respiratory chain, to give peroxynitrite (OONO^−^), which spontaneously decompose to ${\text{NO}}_{2}^{.}$ and hydroxyl radical (.OH). Peroxynitrite is cytotoxic, oxidizes low-density lipoprotein and inhibits mitochondrial function (Radi et al., 2002; Halliwell, 2007). Nitrogen dioxide (NO_2_) derives from the reaction of peroxyl radical and NO, triggers lipid peroxidation and oxidizes ascorbic acid (Patel et al., 1999).

**Figure 1:**
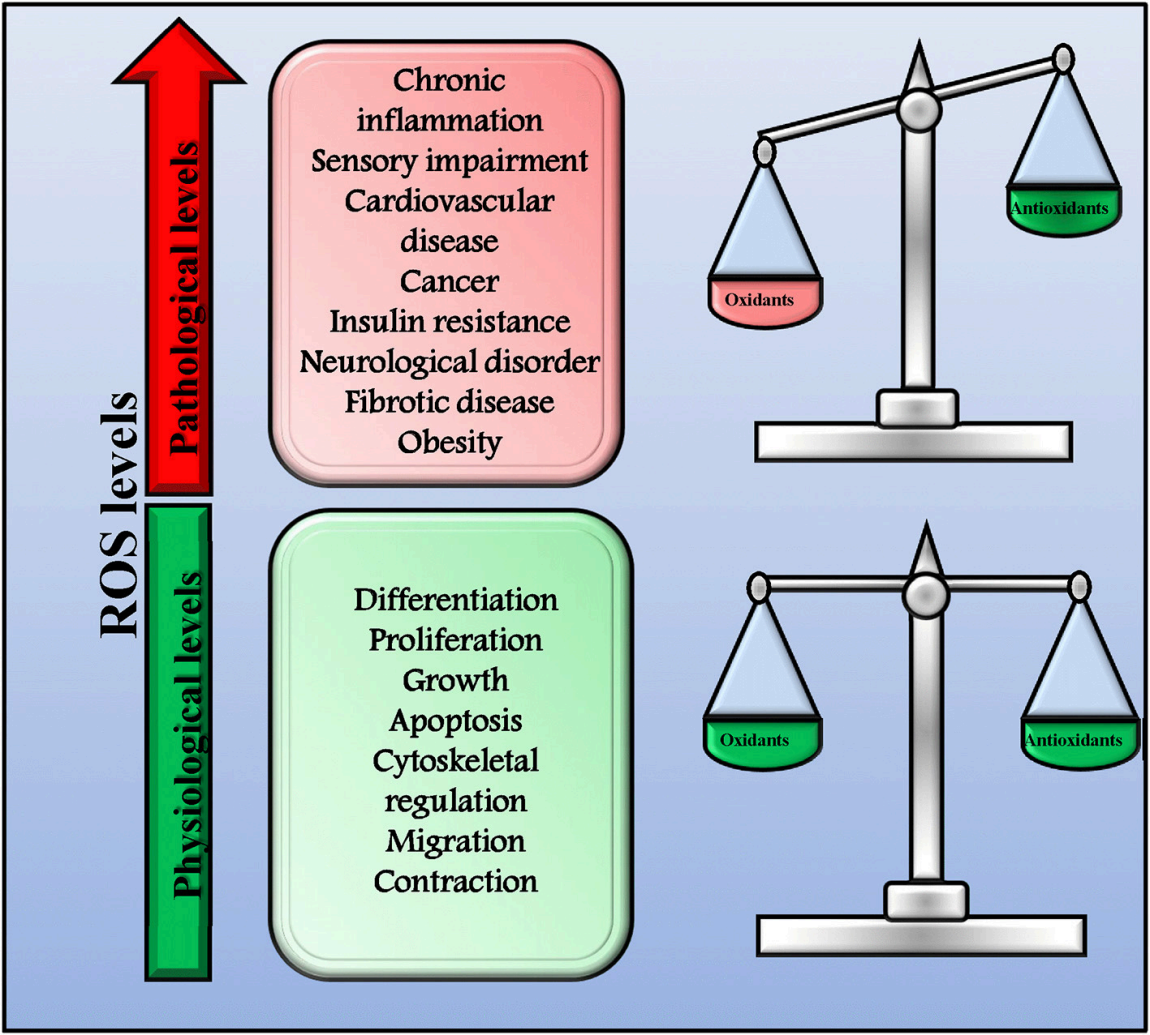
Physiological and pathological ROS levels. In physiological conditions, there is a delicate balance between oxidants and antioxidants that allow cells to conduct their physiological functions and to improve systemic defense mechanisms by inducing an adaptive response. In this conditions ROS production is physiologic and not dangerous. However, when the balance between oxidants and antioxidants is impaired and ROS production increase over the physiological threshold, excessive ROS levels trigger the development of pathologic conditions.

ROS and RNS play a key role in both health and disease acting as signaling molecules (Di Meo et al., 2016). Indeed, they are involved in several physiologic processes (proliferation, growth, differentiation, apoptosis, migration, contraction, and cytoskeletal regulation,) but, when in excess, they also trigger the development of pathologic conditions (chronic inflammation and autoimmune diseases, sensory impairment, cardiovascular diseases, cancer, fibrotic disease, obesity, insulin resistance, neurological disorders, and infectious diseases; Mittler et al., 2011; Sena and Chandel, 2012; Brown and Griendling, 2015). In physiological conditions, a delicate balance between oxidants and antioxidants exists that allow cells to conduct their physiological functions and to improve the systemic defense mechanisms (Figure 1; Ristow and Schmeisser, 2014; Ogun, 2015). However, when this balance is impaired leading to an excessive production of ROS/RNS, oxidative and nitrosative stress occurs and causes extensive cellular damage. This dual effect of ROS has been named mitohormesis, indicating a non-linear dose-response relationship between ROS levels and mortality (Ristow and Schmeisser, 2014).

## The involvement of ROS/RNS in the etiology of hypertension

Hypertension is a complex condition whose etiology depends on several factors (smoking, diet, genetics, family history, pre-existing pathologies) and, in most cases, it is difficult to determine the main cause (“essential hypertension”). Besides the complex etiology of this disease, oxidative and nitrosative stress appear to be a common feature within hypertensive disorders (Harrison et al., 2007; Harrison and Gongora, 2009; Baradaran et al., 2014). Even if it is still debated whether excessive ROS/RNS production is the cause or the consequence of hypertension, several *in vitro* and *in vivo* evidence suggest that ROS/RNS trigger the activation of specific molecular mechanisms which in turn increase blood pressure levels (Ward and Croft, 2006).

### *In vitro* evidence in cultured vascular cells

**Figure 2:**
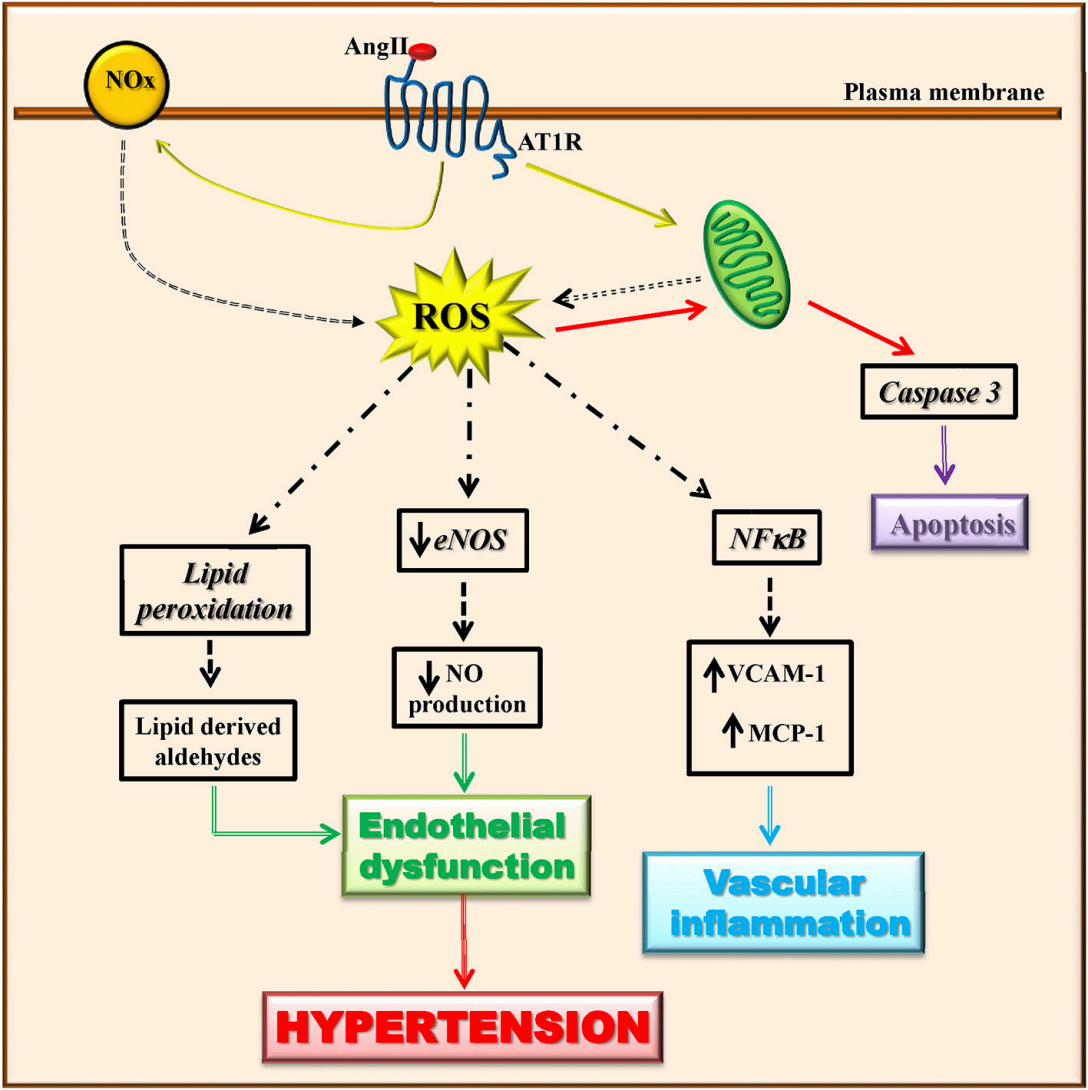
Angiotensin II-dependent ROS production induces hypertension. Angiotensin II induces ROS production through the activation of mitochondrial enzymes and nicotinamide adenine dinucleotide phosphate (NADPH) oxidase (Nox). Angiotensin II-dependent ROS production causes in turn a significant reduction of eNOS activity and NO production, lipid peroxidation, induction of apoptotic signaling, and NFκB activation. These all lead to endothelial dysfunction and vascular inflammation that trigger the development of the hypertensive state.

Vascular cell types (endothelialcells, smooth muscle cells, adventitial fibroblasts, and perivascular adipocytes) are able to produce ROS through the activity of many enzymes (Touyz and Briones, 2011; Kim and Byzova, 2014). Among these latter, mitochondrial enzymes and nicotinamide adenine dinucleotide phosphate (NADPH) oxidase (Nox) are the major sources of ROS in the vascular wall that trigger mitochondrial dysfunction and consequently oxidative stress. Angiotensin II has been shown to induce mitochondrial ROS production through the activation of NADPH oxidase (Doughan et al., 2008; Figure 2). The vascular production of ROS/RNS causes a significant reduction of NO production and eNOS activity (Rodrigo et al., 2011). Indeed, when the levels of superoxide anion increase, nitric oxide is rapidly degraded causing endothelial dysfunction (McIntyre et al., 1999; Touyz and Schiffrin, 2004). The peroxynitrite oxidizes BH4, an important NO synthase cofactor, and inducing an increase of superoxide production leading to the development of oxidative stress (Laursen et al., 2001). Through lipid peroxidation, ROS can also cause the generation of secondary products (lipid-derived aldehydes) that contributes to endothelial dysfunction and hypertension (Cracowski et al., 2002).

In hypertension, ROS affected several processes which in turn trigger endothelial dysfunction (apoptosis, angiogenesis, inflammation). Indeed, in endothelial cells, the increase of ROS production in response to pro-inflammatory and pro-atherosclerotic factors (Ang II, oxLDL, TNFalpha), activates apoptotic events which are prevented by the treatment with antioxidants (Dimmeler and Zeiher, 2000). The pro-apoptotic effects of ROS in endothelial cells derives from the impairment of mitochondrial membrane permeability followed by cytochrome c release and caspase activation (Breitschopf et al., 2000; Lee et al., 2009).

In the endothelium, the expression of some adhesion molecules (vascular cell adhesion molecule-1 and intracellular adhesion molecule-1) is ROS-dependent, suggesting that ROS promote adhesion of inflammatory cells (Marui et al., 1993; Khan et al., 1996).

It has been demonstrated that ROS-dependent angiogenesis is associated with VEGF expression (Kim and Byzova, 2014). Indeed, hydrogen peroxide increases VEGF expression both in vascular smooth muscle cells and in endothelial cells, thus promoting angiogenic responses (Ruef et al., 1997; Chua et al., 1998). ROS also affect the dimerization and autophosphorylation of VEGFR2 in response to VEGF, and subsequent angiogenesis induced by VEGFR2 activation (Colavitti et al., 2002; Ushio-Fukai et al., 2002; Kim and Byzova, 2014). Recent studies also identified novel mechanisms of ROS-dependent angiogenesis which are VEGF-independent (Kim et al., 2013; Kim and Byzova, 2014). Indeed, ROS are involved in the generation of new lipid oxidation products with proangiogenic activities through TLR-2 dependent NFkappaB activation. Also, ROS-dependent NFkappaB activation induces the expression of pro-inflammatory genes (Malinin et al., 2011; Kim et al., 2013; Kim and Byzova, 2014).

### *In vivo* evidence in animal models of hypertension

The involvement of ROS in the etiology of hypertension has been demonstrated in several animal models of hypertension: spontaneously hypertensive rat (Kerr et al., 1999), the angiotensin II-infused rat (Haugen et al., 2000), renovascular hypertension (Lerman et al., 2001), the deoxycorticosterone acetate-salt model (Wu et al., 2001), and obesity-related hypertension (Dobrian et al., 2001). These studies associate oxidative stress with the mechanisms of hypertension, including vascular and organ damage. A further confirmation of ROS involvement in hypertension derives from the finding that in animal models of hypertension the increased ROS production causes endothelial dysfunction that is reversed by SOD (Laursen et al., 1997; Bauersachs et al., 1999; Somers et al., 2000).

### *In vivo* evidence in humans

In smooth muscle cells from arteries of hypertensive patients the treatment with Angiotensin II induces ROS production, as demonstrated by the increase of several parameters that are related to ROS (Touyz and Schiffrin, 2001; Ahmad et al., 2017). Furthermore, in hypertensive patients a strong association exists between blood pressure and the elevated oxidative stress biomarkers such as malondialdehyde, F2-isoprostanes, GSSG, and the DNA oxidation marker 8-oxo-7,8-dihydro-2′-deoxyguanosine (8-oxo-dG) (Rodrigo et al., 2007; Ahmad et al., 2017).

## The antioxidant therapy in hypertension

Given the above discussed involvement of oxidative and nitrosative stress in the etiology of hypertension, the antioxidant therapy seems to be a useful strategy to restore the impaired balance between oxidants and antioxidants in hypertensive conditions. Indeed, the treatment with antioxidants has been successfully used in animal models of hypertension. The oral treatment with Lazaroid, a ROS scavenger, in spontaneously hypertensive rats (SHR) improved NO viability and reduced blood pressure (Vaziri et al., 2000). Similarly, treatment with the antioxidant N-acetylcysteine (NAC) inhibited ROS production and improved NOS activity and accordingly reduced blood pressure (Ahmad et al., 2017). The same results were found also in SHR treated with the xanthine oxidase inhibitor, allopurinol (Mazzali et al., 2001). Moreover, successful results were also obtained by targeting antioxidant peptides to the vasculature to increase the antioxidant effect, reduce vascular resistances and lower BP. For instance, the antioxidant peptide gp91ds affects the assembly of NAD(P)H oxidase and consequently reduces superoxide production (Greig et al., 2010). This peptide was engineered to target vasculature and chronically administered to a preclinical model of endothelial dysfunction and more severe hypertension, the stroke-prone SHR. The treatment significantly improved nitric oxide bioavailability and attenuated the time-dependent and progressive increase in systolic blood pressure (Greig et al., 2010).

Opposite to preclinical models, however, antioxidant strategies for the treatment of hypertension in clinic did not reach the expected success. Indeed, literature is quite discordant on the effect of antioxidant therapy in hypertension as demonstrated by data from clinical trials (Kizhakekuttu and Widlansky, 2010). This could be due to the complexity of this condition. Indeed, while we call hypertensives all patients with blood pressure values above a given threshold (Mancia et al., 2013; Whelton et al., 2017) indeed within this generic definition a much diversified range of phenotypes are included, ranging from the young lean to the obese, to the postmenopausal women or the elderly hypertensives. For each of this phenotype, indeed, it is expected to recognize different etiology, depending on several risk factors (genetics, family history), lifestyles (smoking, diet, sedentary lifestyle), concomitant conditions (chronic kidney disease, diabetes). In each of these phenotypes, the role of oxidants might be different, and therefore diluted within clinical trials that do not select the appropriate patient. Moreover, it is to be considered that the effectiveness of antioxidants can be lowered by the cross-talk with other substances. For instance, it has been shown that Vitamin C alone reduced both systolic and diastolic blood pressure (BP) vs. placebo (Ward et al., 2005) through the down-regulation of NADPH oxidase and up-regulation of eNOS (Briones and Touyz, 2010; Juraschek et al., 2012). However, the same vitamin, in combination with Polyphenols, increases BP, while in combination with other antioxidants (Vitamin E, beta-carotene, and zinc) modestly reduces systolic BP and does not modify diastolic BP (Ardalan and Rafieian-Kopaei, 2014). Furthermore, ineffective dosing regimens and inadequate selection of subjects recruited in the studies could also have affected the effectiveness of the treatment. Table 1 summarize the main common natural antioxidants (e.g., vitamins and mitochondrial related antioxidants), other potential antioxidants (e.g., vitamin D), and anti-hypertensive drugs that also exert antioxidant effects.

**Table 1:** Known and potential antioxidants.

**Antioxidant vitamins**	**Mitochondrial related antioxidants**	**Enzymatic antioxidants**	**Other potential antioxidants**	**Anti-hypertensive drugs**
Vitamin A	Coenzyme Q10	Glutathione peroxidase	Vitamin D	Propanolol
Vitamin C	Acetyl-L-Carnitine	Catalase	Glutamate	Nebivilol
Vitamin E	α-Lipoic Acid	Superoxide dismutase	N-acetylcysteine	Carvedilol
L-Arginin			Sour milk	Celiprolol
Flavonoids			Garlic	Amlodipine
				Enalapril

Among natural antioxidants, here we focused on Vitamin D, whose levels has been recently associated with hypertension, since it has great potentiality to be used for therapeutic treatments. Among the other antioxidants listed in Table 1, we discuss the ability of some anti-hypertensive drugs to reduce oxidative stress. This latter property of anti-hypertensive drugs enforces the proof of concept about the key role of oxidative stress in the development and progression of hypertensive states and the benefit of antioxidants as therapeutic strategy.

### Vitamin D

**Figure 3:**
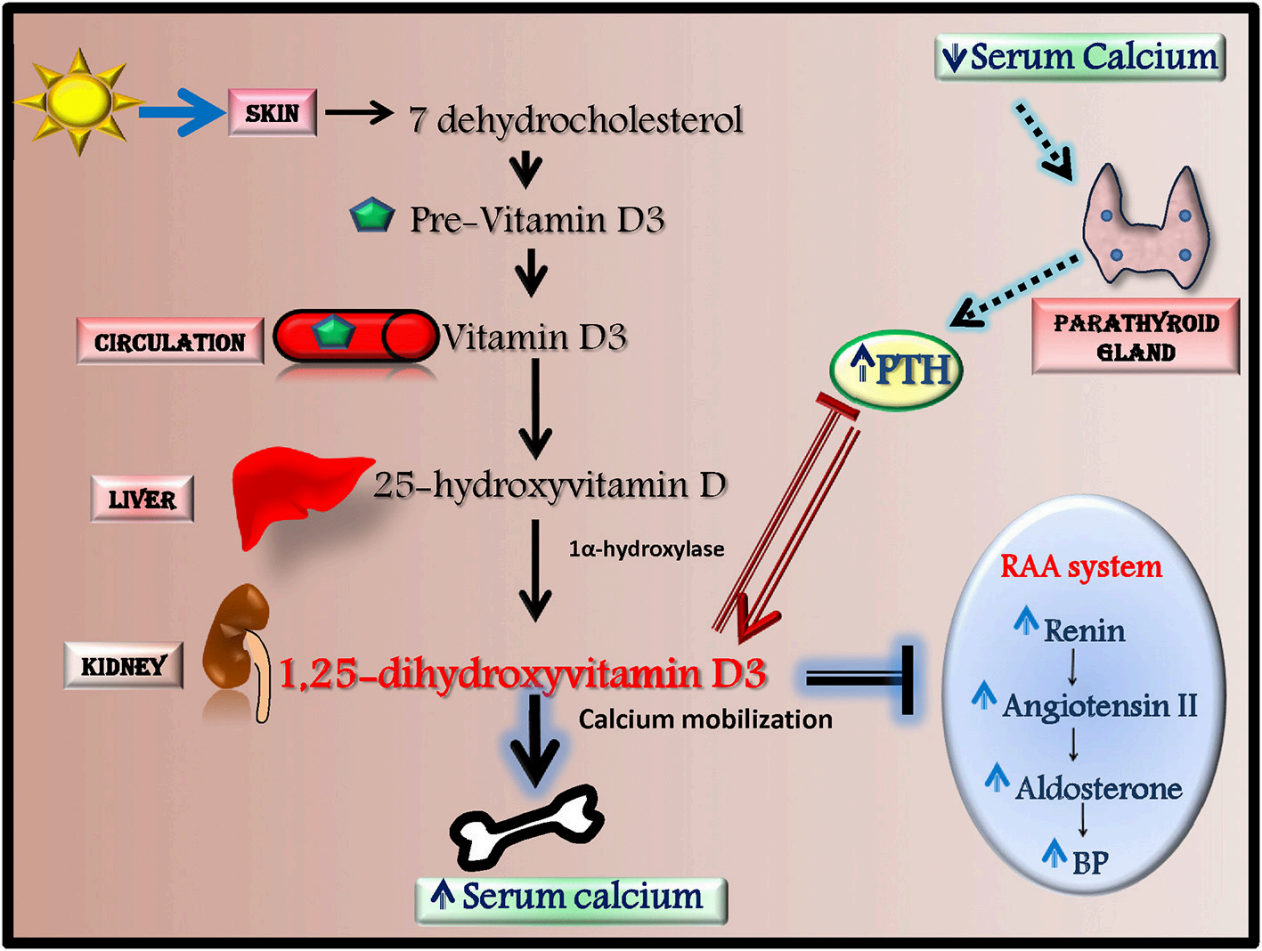
Vitamin D synthesis and effects. Most vitamin D is naturally synthesized in the skin from 7-dehydrocholesterol in response to ultraviolet radiation. 7-dehydrocholesterol is converted to Pre-Vitamin D3 that through the circulation reach the liver where it is metabolized to 25-hydroxyvitamin D (25(OH)D). This latter is then converted in the kidney by 1α-hydroxylase into 1,25-dihydroxyvitamin D3, the biologically active agonist for the Vitamin D receptor. The synthesis of 1,25-dihydroxyvitamin D3 is mainly regulated by PTH and serum calcium levels. 1,25-dihydroxyvitamin D3 has a several effects since it increases serum calcium levels by inducing calcium mobilization from bone, decreases renin-angiotensin-aldosterone system (RAAS) activity and inhibits PTH production.

Among antioxidants, Vitamin D is recently emerging as anti-hypertensive effector through the activation of antioxidant mechanisms. In human, most vitamin D (~80%) is naturally synthesized in the skin from 7-dehydrocholesterol in response to ultraviolet (UV) B radiation but it can also derive from dietary sources. Vitamin D is metabolized in the liver to 25-hydroxyvitamin D (25(OH)D) that is converted by 1α-hydroxylase into 1,25-dihydroxyvitamin D3, the biologically active agonist for the Vitamin D receptor (VDR) (Figure 3; Chen et al., 2015). Serum levels of Vitamin D are regulated by calcium homeostasis and parathyroid hormone (PTH) level since low calcium and high PTH levels induce Vitamin D synthesis by increasing 1α-hydroxylase activity (Chen et al., 2015).

A large part of western population is thought to have a Vitamin D deficiency/insufficiency, which has been associated with an increased risk for cardiovascular diseases (McGreevy and Williams, 2011; Tamez et al., 2013). The reason for this deficiency can be probably due to a decreased exposure to sun as a prevention for melanoma (Holick, 2007), although nutritional aspects are also being considered and posed a the base of replacement therapy strategies.

Recently, an association between low Vitamin D serum levels and hypertension have been suggested (Ullah et al., 2010; Kota et al., 2011). Indeed, 1-alpha-hydroxylase deficient mice, which cannot synthesize Vitamin D3, develop high blood pressure and left ventricular hypertrophy (Zhou et al., 2008). Vitamin D can affect blood pressure through several mechanisms. Indeed, in both animals and humans it has been shown that vitamin D decreases renin-angiotensin-aldosterone system (RAAS) activity (Li et al., 2002; Tomaschitz et al., 2010), modulates endothelial function (Wong et al., 2010; Pittarella et al., 2015; Molinari et al., 2018) and regulates vascular oxidative stress (Argacha et al., 2011).

Clinical studies demonstrated an inverse, dose-response relationship between plasma Vitamin D3 concentration and blood pressure or renin activity in both normotensive and hypertensive patients (Nigwekar and Thadhani, 2013; Grubler et al., 2017). High levels of Vitamin D in humans, for instance, are associated with lower blood pressure (Vimaleswaran et al., 2014). All these reports suggest that Vitamin D levels are associated with BP also in humans. Based on such findings, it is likely to believe that Vitamin D supplementation could be an effective therapy for hypertension. This hypothesis was confirmed in animal models of hypertension. Indeed, Vitamin D supplementation ameliorates pathological right ventricular hypertrophy in rats with pulmonary hypertension (Tanaka et al., 2017) and reduces blood pressure levels in SHR rats (Wong et al., 2010). Accordingly, several clinical trials show the effectiveness of natural vitamin D, Vitamin D3 or its analog supplementation on BP levels in those patients with essential hypertension that is dependent on Vitamin D-deficiency (Kimura et al., 1999; Pfeifer et al., 2001; Judd et al., 2010; Goel and Lal, 2011; Bernini et al., 2013; Forman et al., 2013; Carrara et al., 2014; Mozaffari-Khosravi et al., 2015). Vitamin D supplementation therapy also in pregnancy is able to reduce the incidence of gestational hypertension/preeclampsia (Behjat Sasan et al., 2017). Moreover, Vitamin D have beneficial effects on BP also in patients affected by other pathologies, such as type 2 diabetes (de Paula et al., 2017).

However, other randomized controlled trials show that Vitamin D supplementation results ineffective as anti-hypertensive agent (Li et al., 2004; Michos and Melamed, 2008; Beveridge et al., 2015; Grubler et al., 2017; Wu and Sun, 2017). Thus, literature seems to be quite discordant on the effectiveness of Vitamin D supplementation in the treatment of the hypertensive condition. However, this discrepancy could be dependent on several variables in study population (Vitamin D-deficiency levels, gender, ethnicity, BP levels, age, parathormone levels). Indeed, a recent study show that the relationship between serum levels of Vitamin D and BP differs according to ethnicity and gender with a significant inverse association among non-hispanic whites (NHW) and females, NHW females and non-hispanic black females (Vishnu and Ahuja, 2017). Data from this study suggest a non-linear relationship between Vitamin D and hypertension with significant decline in hypertension only up to a physiological level of Vitamin D that is different depending on race/ethnicity and gender (Vishnu and Ahuja, 2017). Among the putative mechanisms involved in the association between Vitamin D deficiency and increased blood pressure levels, anti-oxidant effects of Vitamin D have been implicated. Nevertheless, it has also to consider that Vitamin D is inversely correlated with the calcium modulator paratohormone (PTH). The vascular effects of such hormone, as well as the mechanisms associated with the reduced kidney function are both possible mechanisms of increased vascular resistance and blood volume, two determinants of hypertension. Indeed, increased PTH has been demonstrated to correlates better than Vitamin D deficiency with blood pressure and cardiovascular risk, including hypertension, in a large population in Southern Italy (Pascale et al., 2018) suggesting that also PTH levels could be a discriminating parameter in the selection of patients that could be sensitive to Vitamin D supplementation. Thus, future researches on this issue should take into account these parameters and, accordingly, identify an ideal population which result more sensitive to this kind of treatment.

### Anti-hypertensive drugs with antioxidant properties

To date, several molecules have been discovered that are effective anti-hypertensive drugs with antioxidant properties. Indeed, some beta-blockers, apart from their ability to inhibit adrenaline/noradrenaline dependent activation of beta adrenergic receptors (Iaccarino et al., 2006; Sorriento et al., 2011; Galasso et al., 2013), are also able to reduce oxidative stress. Among them, Propanolol, Nebivilol, Carvedilol, and Celiprolol are the most studied (Yao et al., 2008). Propranolol inhibits oxidative stress and reduces tissue lipid peroxidation (Mak and Weglicki, 1988; Yao et al., 2008). Carvedilol reduces lipid peroxidation in patients with heart failure by acting as a free radical scavenger (Kukin et al., 1999; Yao et al., 2008). Celiprolol reduces superoxide anions generation in patients with essential hypertension and improves endothelial function (Mehta et al., 1994; Kobayashi et al., 2001; Yao et al., 2008). However, this antioxidant effects are not a common feature of all beta-blockers since it has been shown that Atenolol has no effect on ROS production in endothelial cells (Fratta Pasini et al., 2005).

At the molecular level, the beta-blocking effect is itself important to reduce ROS production by blocking catecholamines that are known to induce oxidative stress in the myocardium. Furthermore, some beta-blockers have also direct antioxidant effects which are different depending on the modulation of specific intracellular signaling.

Indeed, Nebivolol exerts its effects by increasing NO levels, NOS activity, and expression of eNOS, as well as by reducing ROS production and Nox expression (Wang et al., 2017). Carvedilol inhibits 4-hydroxy-2-nonenal (HNE)-induced intracellular Ca^2+^overload (Nakamura et al., 2011). Celiprolol significantly suppresses BP levels and ameliorates hypoxia-induced LV remodeling in mice, by restoring eNOS expression via stimulation of PI3K-AKT signaling pathway (Kobayashi et al., 2003; Nishioka et al., 2013).

Besides these beta-blockers, also Amlodipine, a calcium channel blocker, shares the same anti-hypertensive and antioxidant properties. Indeed, Amlodipine is able to decrease blood pressure as well as oxidative stress as shown by a decrease of malondialdehyde and an increase of Na^+^ K^+^ ATPase and SOD levels in essential hypertensive patients (Mahajan et al., 2007). This effect is further increased by Vitamin C supplementation (Mahajan et al., 2007).

Furthermore, Enalapril, an ACE-inhibitor, reduces the expression of oxidant stress markers and antioxidant enzymes in the heart and kidney of SHR rats (Chandran et al., 2014; Yusoff et al., 2017) and of diabetic rats (de Cavanagh et al., 2001). Also in hypertensive patients, 3 months of Enalapril therapy are beneficial to prevent oxidative stress compared with Atenolol treated patients (Deoghare and Kantharia, 2013). Similarly, the antioxidant beneficial effects on vascular biology, including nitrix oxide availability, has been demonstrated for SH- containing ACE inhibitors (Captopril, Lisinopril, zofenopril) due to the free radical scavenging properties of the tiol residues contained in the drug sequence (Chopra et al., 1990; Buikema et al., 2000; Donnarumma et al., 2016).

## Conclusions and future directions

Several diseases have been associated with oxidative stress suggesting that this latter could be a trigger for diseases and that antioxidant therapy could be an effective therapeutic treatment. However, while basic research and pre-clinical studies support this point of view, clinical studies still produce controversial results. This could probably be dependent on the pathophysiological complexity of ROS/RNS signaling in humans with comorbidities (Pagliaro and Penna, 2015, 2017; Egea et al., 2017). Here, in particular, we have discussed about the role of oxidative stress in the development and progression of hypertensive states even if the idea that antioxidant therapy is effective against this disease by inhibiting or destroying free radicals is not accepted yet. Indeed, the promising results in pre-clinical model of hypertension are not always support by data from patients. A great discrepancy exists among results from different clinical trials. Actually, limitations to the effectiveness of antioxidant therapy in the management of hypertension could be due to numerous variables. First of all, the half-life of the particular antioxidant administered affects its effectiveness in long-term treatments. Moreover, the cross-talks with other substances in some cases reduce the anti-hypertensive effects. Finally, the inadequate homogeneity of patients characteristics in study population is probably the most important limitation of clinical trials. To date, the use of anti-hypertensive drugs with antioxidants properties seems to be the most effective treatment in the management of hypertension since they are able to reduce blood pressure by affecting molecular mechanisms which are involved in the regulation of both vascular function and oxidative state. Despite the discordant results of clinical trials, Vitamin D supplementation could also be a promising therapeutic treatment for hypertension that is worthwhile to further investigate considering not only the rate of Vitamin D deficiency, but also PTH levels, as discriminating factors in the selection of patients. For the future improvement of antioxidant therapy the above proposed potential limitations should be taken into account. Moreover, further studies are needed to better clarify the sources and targets of ROS/RNS and their harmful or beneficial roles, the specific molecular mechanisms and their cross-talks, and to identify the ideal patient which could be sensitive to specific antioxidant therapies.

## Author contributions

DS, ND, BT, and GI conceived and designed the work, drafted the work and revisited it critically.

### Conflict of interest statement

The authors declare that the research was conducted in the absence of any commercial or financial relationships that could be construed as a potential conflict of interest.
